# Supplementary material for: Toll-like receptor mediated inflammation directs B cells towards protective antiviral extrafollicular responses
Source: Nat Commun. 2023 Jul 5;14:3979. doi: 10.1038/s41467-023-39734-5 (PMC10322839; doi:10.1038/s41467-023-39734-5)
Supplement: Supplementary file 1 — Supplementary Information [file 41467_2023_39734_MOESM1_ESM.pdf]

Supplemental Materials for:

Toll-like receptor mediated inflammation directs B cells towards protective antiviral extrafollicular responses

Jonathan H. Lam[1,2] and Nicole Baumgarth[1,2,3,4]

[1]Graduate Group in Immunology, [2]Center for Immunology and Infectious Diseases, [3]Dept.

Pathology, Microbiology and Immunology, University of California Davis, Davis, USA

Supplemental Figures 1-9

[4]Correspondence

Nicole Baumgarth DVM PhD

Dept Molecular Microbiology and Immunology

Johns Hopkins Bloomberg School of Public Health

615 N Wolfe St, E4135

P: 410 614 2718

Email: nbaumga3@jhmi.edu

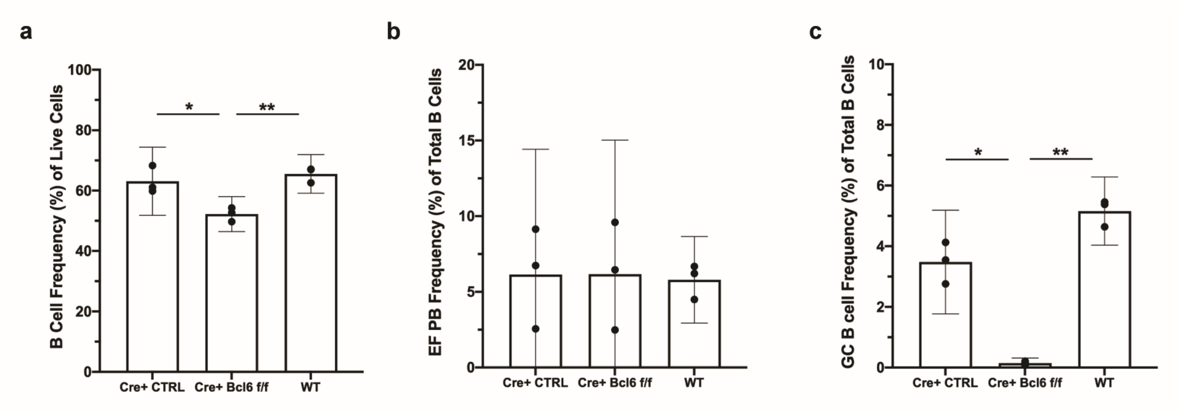

Supplemental Figure 1. EFR are not generated through GC reactions. WT, Mb-1 Cre+ control, and Mb-1 Cre+ Bcl6 f/f mice (n=3) were infected with 10 PFU A/PR8 and medLNs were analyzed at 10 dpi. **(a)** B cell frequency of total cells. **(b)** EF PB frequency of total B cells. **(c)** GC B cell frequency of total B cells. Data represent mean ± 95% CI. Statistical significance determined by one-way ANOVA and two-tailed Student's t-test with Welch's correction. *: $p<0.05$, **: $p<0.01$. Source data are provided as a Source Data file.

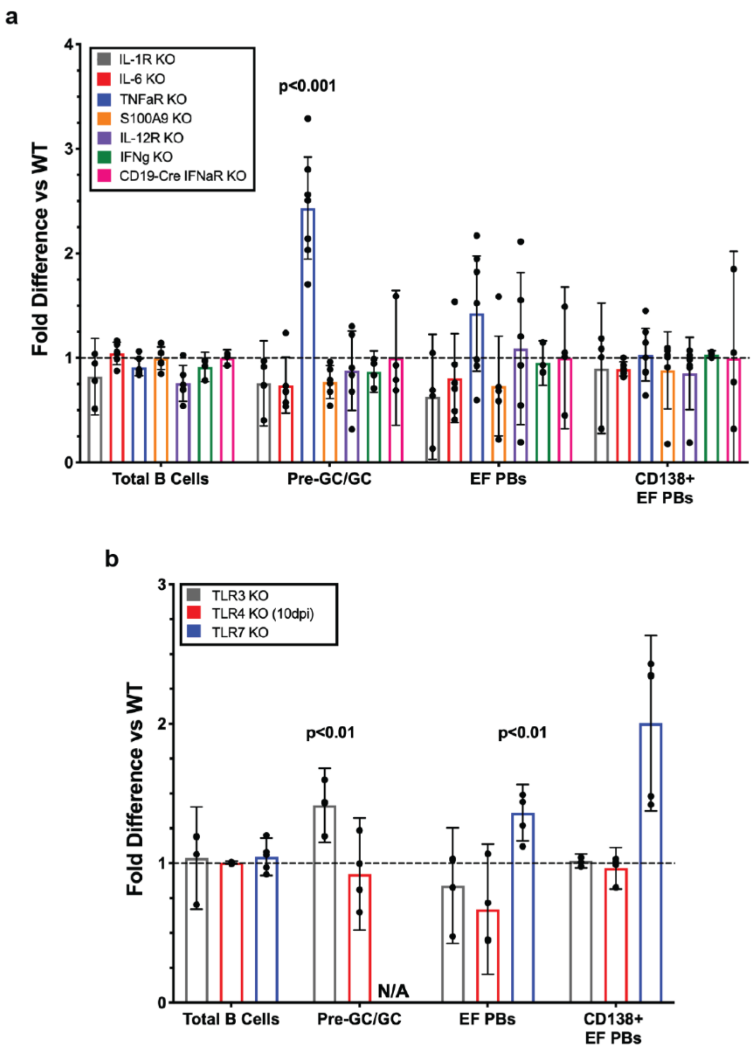

Supplemental Figure 2. Lack of single cytokine or TLR pathway does not affect EFR to influenza. Knockout and WT mice were infected with 10 PFU A/PR8 and medLNs were collected at 7 days post-infection (dpi). Shown are fold-differences in major B cell subsets in C57BL/6 WT and indicated gene-targeted mice (n=4-7) (**a**), as well as single TLR-knockouts (n=4-5), (**b**). Data represent mean ± 95% CI of two independent experiments. Statistical significance determined by one-way ANOVA and two-tailed Student’s t-test with Welch’s correction. P-values indicated on charts. Source data are provided as a Source Data file.

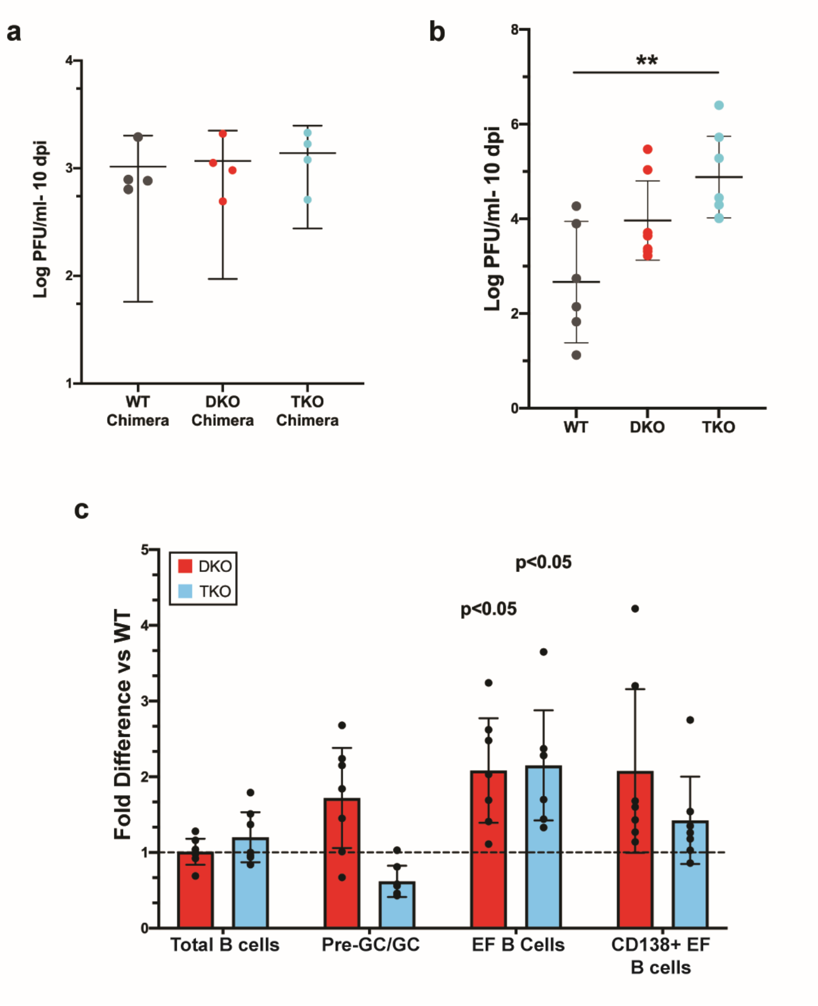

Supplemental Figure 3. **Passive protection with TKO serum and late-stage viral loads and EF responses.** (**a**,**b**) Viral loads as analyzed by viral qRt-PCR on lung homogenates at 10 dpi of B cell-specific chimera (n=4) (**a**) and global knockout (n=6-7) (**b**) mice. **c)** Fold-differences of B cell subsets in DKO, TKO and WT mice (n=7) at 10 dpi. Data represent mean ± 95% CI of two independent experiments. Statistical significance determined by one-way ANOVA and two-tailed Student's t-test with Welch's correction. *: p<0.05 (or shown) **: p<0.01. Source data are provided as a Source Data file.

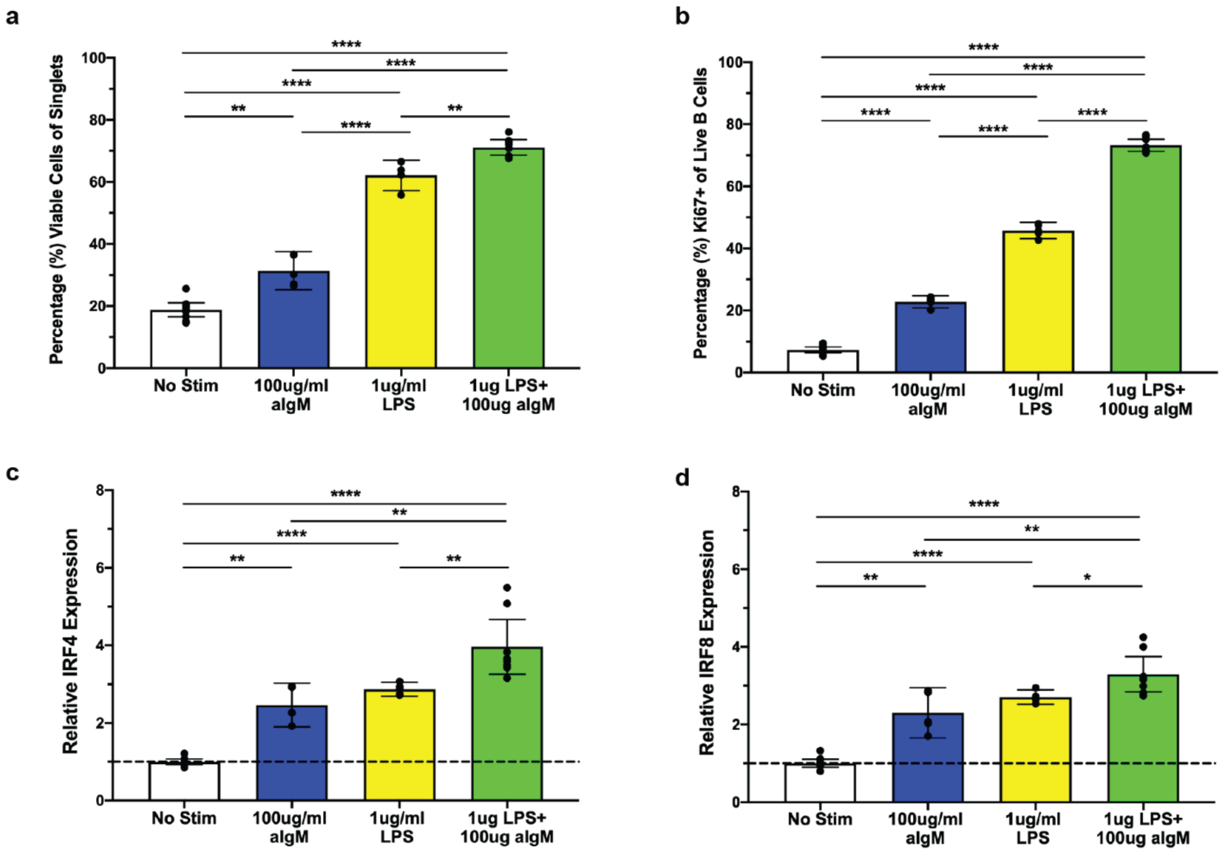

Supplemental Figure 4. BCR and TLR4 stimulation have additive effects on cell viability, proliferation, and activation phenotype. Pooled and enriched splenic and lymph node B cells from C57BL/6 mice (n=2) were cultured with pulsed 100 µg/ml anti-IgM for 3h, and/or sustained 1µg/ml LPS, then assessed for (**a**) cell viability, (**b**) proliferation, (**c**) IRF4 and (**d**) IRF8 after 48h. Data represent mean ± 95% CI of two independent experiments. Data contain n=5-10 total replicates per group. Statistical significance determined by one-way ANOVA and two-tailed Student's t-test with Welch's correction. *: $p<0.05$ **: $p<0.01$, ****: $p<0.0001$. Source data are provided as a Source Data file.

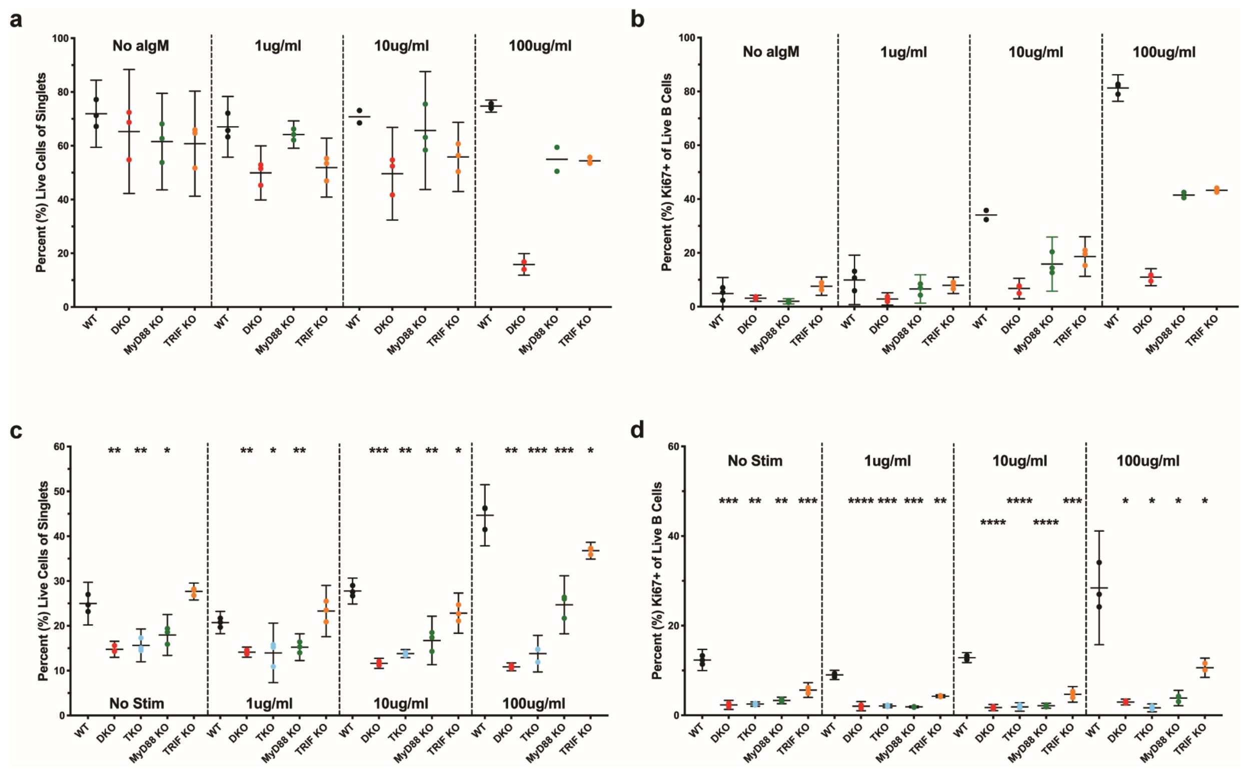

Supplemental Figure 5. MyD88 and TRIF non-redundantly regulate B cell viability and proliferation in response to BCR stimulus. Pooled splenic/LN B cells from WT, DKO, MyD88 KO, and TRIF KO mice (n=2-3) were negatively enriched (>98%) and pulsed with graded anti-IgM for 3 hours, then stimulated with CD40L and BAFF for 48 hours and assessed for **(a)** viability and **(b)** proliferation. **(c,d)** Cells as in **(a)** were pulsed with graded doses anti-IgM for 3 hours followed by incubation in complete medium only for 48h, then assessed for (**c**) cell viability and (**d**) proliferation. Data represent mean ± 95% CI. Statistical significance determined by one-way ANOVA and two-tailed Student's t-test with Welch's correction. *: p<0.05 **: p<0.01 ***: p<0.001, ****: p<0.0001. Source data are provided as a Source Data file.

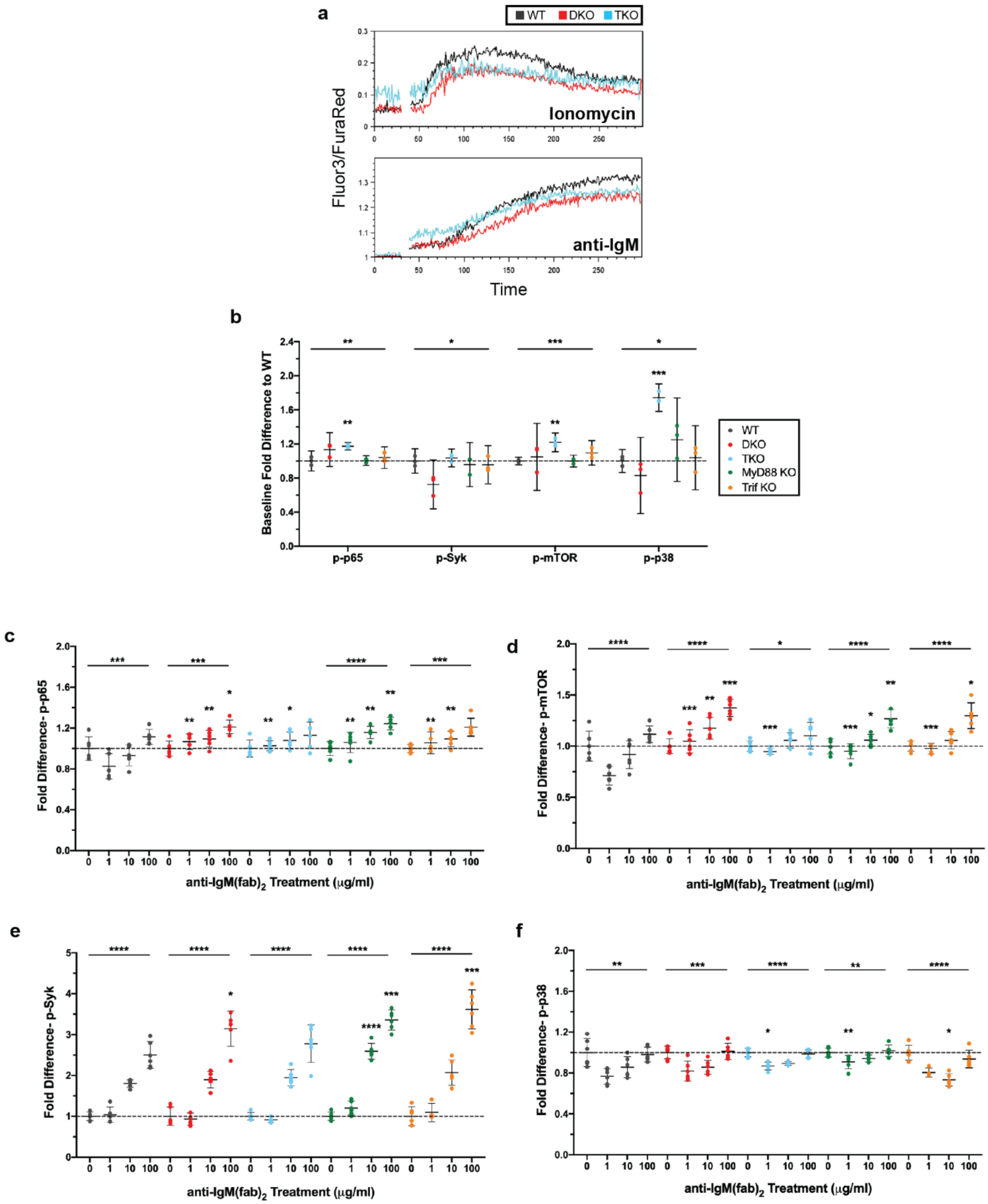
a
WT
DKO
TKO
Fluor3/FuraRed
Ionomycin
anti-IgM
Time
b
Baseline Fold Difference to WT
WT
DKO
TKO
MyD88 KO
Trif KO
p-p65
p-Syk
p-mTOR
p-p38
c
Fold Difference- p-p65
anti-IgM(fab)2 Treatment (μg/ml)
d
Fold Difference- p-mTOR
anti-IgM(fab)2 Treatment (μg/ml)
e
Fold Difference- p-Syk
anti-IgM(fab)2 Treatment (μg/ml)
f
Fold Difference- p-p38
anti-IgM(fab)2 Treatment (μg/ml)

Supplemental Figure 6. TLR-signaling deficient B cells have nominal immediate activation and functional downstream effector protein activation. (**a**) Calcium flux of BCR-independent (iono) and BCR-mediated, anti-IgM$(Fab)_2$ activation in purified WT, DKO, and TKO B cells. (**b,c**) Pooled splenic/LN B cells from WT, DKO, TKO, MyD88 KO, and TRIF KO mice (n=2-3) were negatively enriched (>98%) and stimulated with graded indicated doses anti-IgM$(Fab)_2$ for 30 minutes, then assessed for phosphorylated, intracellular proteins. (**b**) Baseline fold-difference in phosphorylation between WT and knockout B cells (3 replicates). (**c-f**) Fold-changes in protein phosphorylation of NFkB p65 (**c**), mTOR (**d**), Syk (**e**), and MAPK p38 (**f**) relative to non-stimulated conditions for each strain. Data represent mean ± 95% CI of two independent experiments. Data contain n=3 (**b**) or n=6 (**c-f**) total replicates per group. Statistical significance determined by one-way ANOVA (upper stars comparing treatments within strain) and two-tailed Student's t-test with Welch's correction (lower stars comparing KOs to respective WT controls). *: $p<0.05$ **: $p<0.01$ ***: $p<0.001$, ****: $p<0.0001$. Source data are provided as a Source Data file.

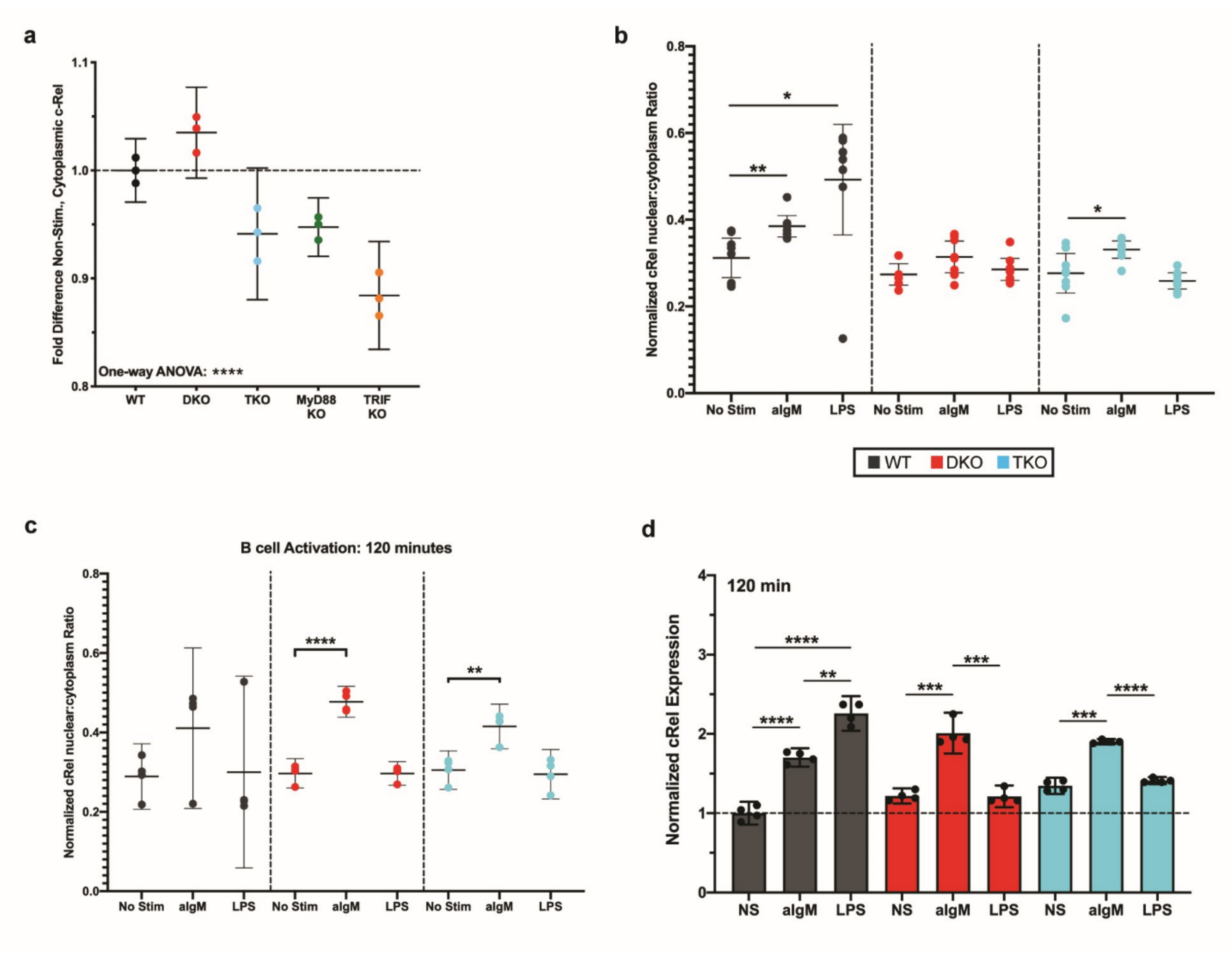

Supplemental Figure 7. Immediate activation of c-Rel after BCR stimulation is defective in TLR-signaling deficient B cells. (**a**) Fold difference between WT and TLR-null cytoplasmic c-Rel in B cells in non-stim conditions as determined by flow cytometry (n=3). (**b-c**) Shown are the ratios of nuclear vs cytoplasmic c-Rel in pooled, negatively-enriched B cells from WT, DKO, and TKO mice (n=2-3) as determined by ELISA on nuclear versus cytoplasmic protein extracts at 60 minutes **(b)** and 120 minutes **(c)** after anti-IgM or LPS stimulation. (**d**) Total c-Rel expression after 120-minute anti-IgM or LPS stimulation as assessed by flow cytometry. Data represent mean ± 95% CI of two independent experiments. Data contain n=8 (**b**) and n=4 (**c,d**) total replicates per group. Statistical significance determined by one-way ANOVA (a, intra-strain comparisons in b-d) and two-tailed Student's t-test with Welch's correction (b-d). *: $p<0.05$ **: $p<0.01$ ***: $p<0.001$, ****: $p<0.0001$. Source data are provided as a Source Data file.

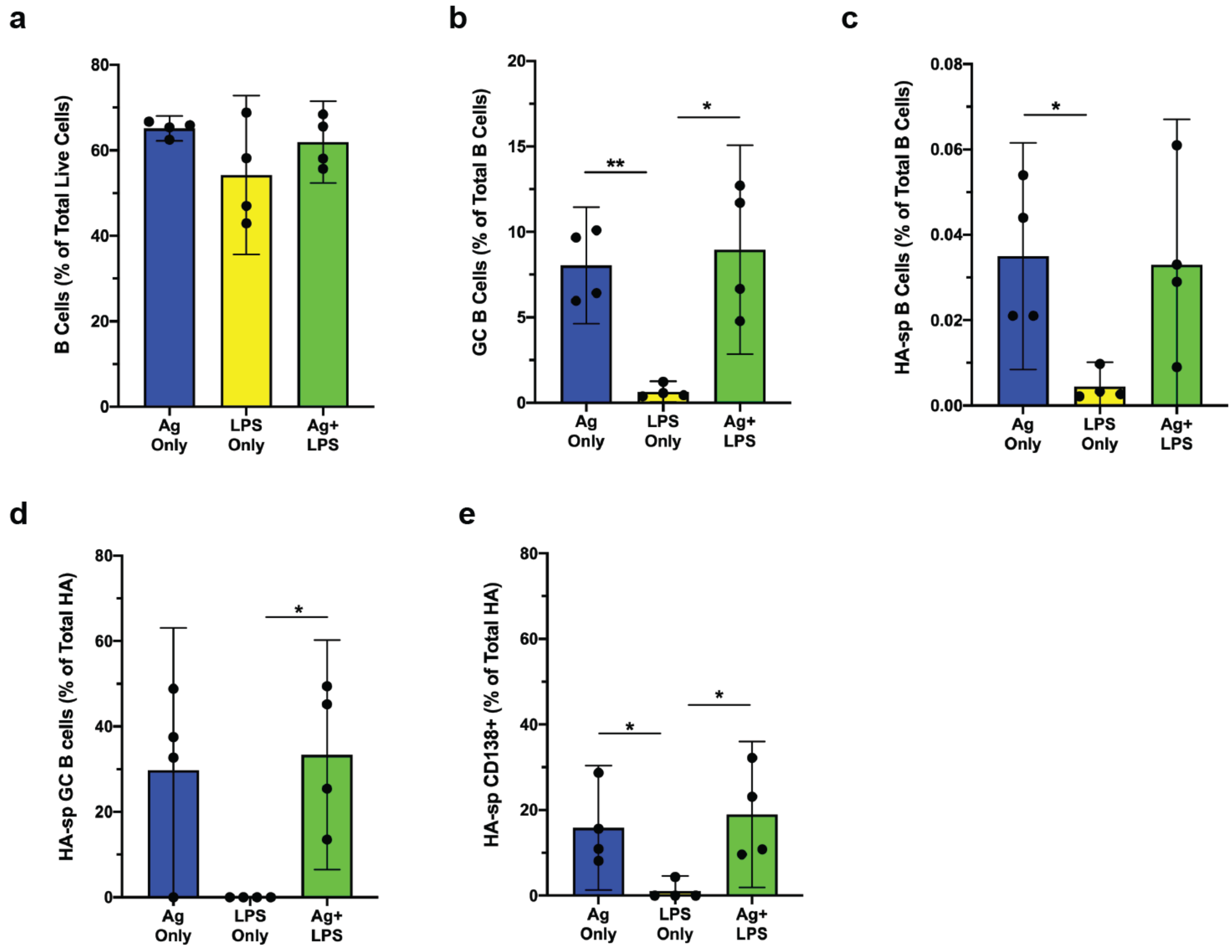

Supplemental Figure 8. EFR induction by LPS boosting has no detrimental effect on GC development. C57BL/6 mice (n=4) were immunized and boosted s.c. as described in Figure 8a, followed by analysis of draining LNs on 14 days post-immunization. (**a)** Frequency of Total B cells. **(b)** Frequency of GC B cells. (**c**) Frequency of HA-specific B cells. (**d**) Frequency of HA-specific GC B cells. (**e**) Frequency of HA-specific CD138+ B cells. Graphs are representative of two experiments (n=4). Data represent mean ± 95% CI of two independent experiments. Statistical significance determined by one-way ANOVA and two-tailed Student's t-test with Welch's correction. *: $p<0.05$ **: $p<0.01$. Source data are provided as a Source Data file.

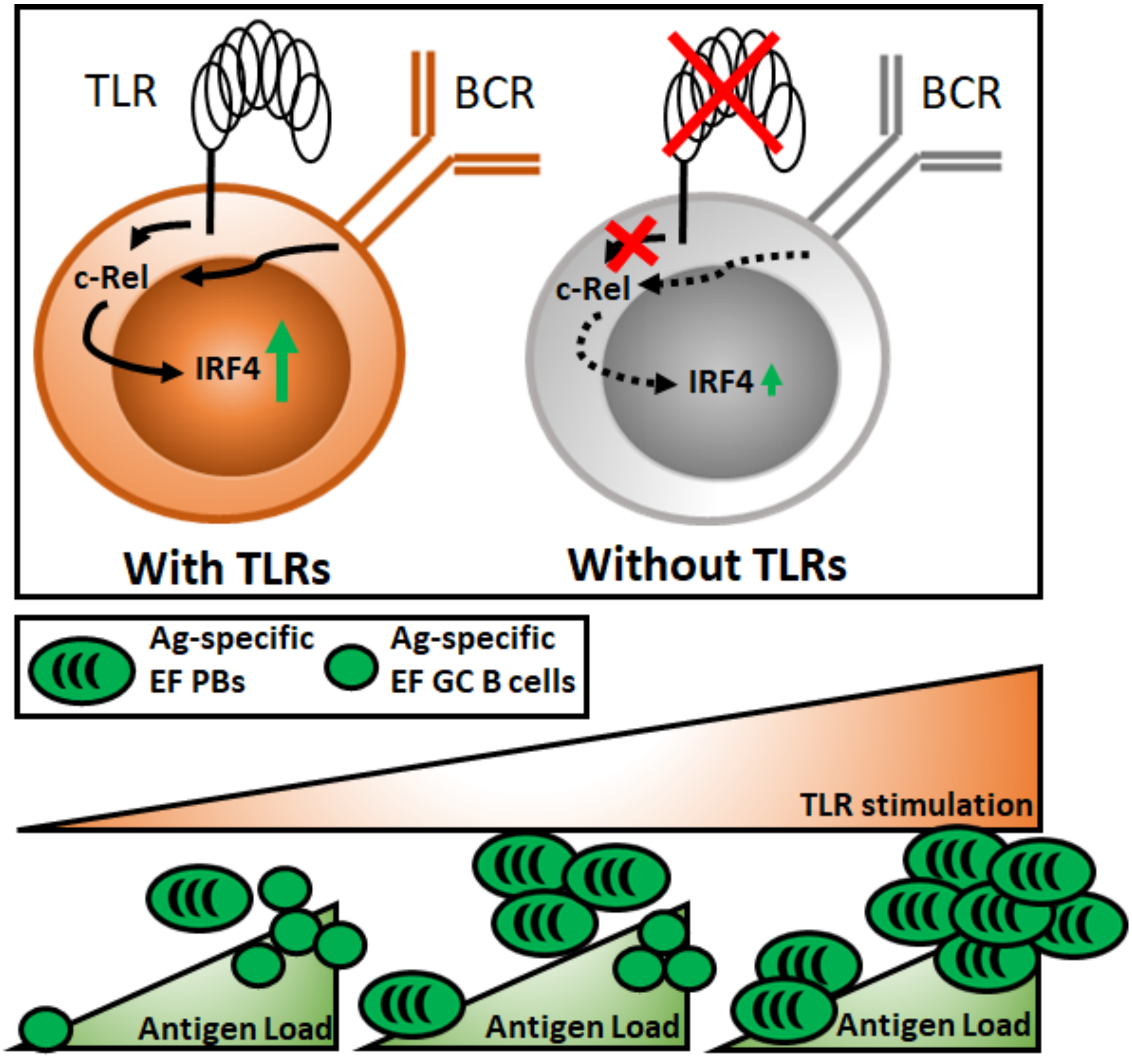

Supplemental Figure 9. Model of TLR-mediated induction of B cell differentiation and EFRs. (Top) With a functional TLR axis either through MyD88/TRIF or TLR2/4/unc93b, BCR stimulation leads to efficient c-Rel nuclear localization and sustained upregulation of IRF4. Without a functional TLR axis, BCR signaling leads to defective c-Rel localization, lack of long-term c-Rel expression, and a lack of sustained IRF4 induction and upregulation. (Bottom) During infection or immunization, TLR agonism polarizes antigen-specific B cells towards EFR and away from the GC response when antigen is limiting or in excess, resulting in early and protective antigen-specific antibody generation.
