# Supplementary material for: Toll-like receptor mediated inflammation directs B cells towards protective antiviral extrafollicular responses
Source: Nat Commun. 2023 Jul 5;14:3979. doi: 10.1038/s41467-023-39734-5 (PMC10322839; doi:10.1038/s41467-023-39734-5)
Supplement: Supplementary file 3 — Reporting Summary [file 41467_2023_39734_MOESM3_ESM.pdf]

nature portfolio

Corresponding author(s): Nicole Baumgarth

Last updated by author(s): 06/23/2023

Reporting Summary

Nature Portfolio wishes to improve the reproducibility of the work that we publish. This form provides structure for consistency and transparency in reporting. For further information on Nature Portfolio policies, see our Editorial Policies and the Editorial Policy Checklist.

Statistics

For all statistical analyses, confirm that the following items are present in the figure legend, table legend, main text, or Methods section.

n/a	Confirmed	
☐	☒	The exact sample size (*n*) for each experimental group/condition, given as a discrete number and unit of measurement
☐	☒	A statement on whether measurements were taken from distinct samples or whether the same sample was measured repeatedly
☐	☒	The statistical test(s) used AND whether they are one- or two-sided<br>*Only common tests should be described solely by name; describe more complex techniques in the Methods section.*
☒	☐	A description of all covariates tested
☐	☒	A description of any assumptions or corrections, such as tests of normality and adjustment for multiple comparisons
☐	☒	A full description of the statistical parameters including central tendency (e.g. means) or other basic estimates (e.g. regression coefficient) AND variation (e.g. standard deviation) or associated estimates of uncertainty (e.g. confidence intervals)
☐	☒	For null hypothesis testing, the test statistic (e.g. *F*, *t*, *r*) with confidence intervals, effect sizes, degrees of freedom and *P* value noted<br>*Give P values as exact values whenever suitable.*
☒	☐	For Bayesian analysis, information on the choice of priors and Markov chain Monte Carlo settings
☒	☐	For hierarchical and complex designs, identification of the appropriate level for tests and full reporting of outcomes
☒	☐	Estimates of effect sizes (e.g. Cohen's *d*, Pearson's *r*), indicating how they were calculated

Our web collection on statistics for biologists contains articles on many of the points above.

Software and code

Policy information about availability of computer code

Data collection	Flow cytometry data was collected with BD FACSDiva, ELISPOT images were collected using an AID Elispot Reader and quantified using AID Elispot 7.0, ELISA data were collected using Molecular Devices SpectraMax M5 and quantified using Softmax Pro 7. rtPCR data was collected with Applied Biosystems QuantStudio 6 Flex and quantified using QuantStudio Realtime PCR.
Data analysis	Flow cytometry data were analyzed using FlowJo v10. Weight loss, viral loads, flow cytometry, ELISPOT, and ELISA data were analyzed using Microsoft Excel and Graphpad prism v8.

For manuscripts utilizing custom algorithms or software that are central to the research but not yet described in published literature, software must be made available to editors and reviewers. We strongly encourage code deposition in a community repository (e.g. GitHub). See the Nature Portfolio guidelines for submitting code & software for further information.

Data

Policy information about availability of data

All manuscripts must include a data availability statement. This statement should provide the following information, where applicable:

- Accession codes, unique identifiers, or web links for publicly available datasets
- A description of any restrictions on data availability
- For clinical datasets or third party data, please ensure that the statement adheres to our policy

All data supporting the findings of this study are available within the paper, its Supplementary Information, and source data file.

Research involving human participants, their data, or biological material

Policy information about studies with human participants or human data. See also policy information about sex, gender (identity/presentation), and sexual orientation and race, ethnicity and racism.

Reporting on sex and gender	N/A
Reporting on race, ethnicity, or other socially relevant groupings	N/A
Population characteristics	N/A
Recruitment	N/A
Ethics oversight	N/A

Note that full information on the approval of the study protocol must also be provided in the manuscript.

Field-specific reporting

Please select the one below that is the best fit for your research. If you are not sure, read the appropriate sections before making your selection.

☒ Life sciences ☐ Behavioural & social sciences ☐ Ecological, evolutionary & environmental sciences

For a reference copy of the document with all sections, see nature.com/documents/nr-reporting-summary-flat.pdf

Life sciences study design

All studies must disclose on these points even when the disclosure is negative.

Sample size	Sample size was determined by an initial experiment of n=2-4 in each group (e.g WT vs KO) then repeated to ensure phenotypic differences were reproducible and statistically significant.
Data exclusions	No data were excluded from analyses.
Replication	Nearly all experiments conducted were explicitly replicated at least twice. Experiments involving c-Rel nuclear localization were additionally replicated using two different readouts (flow cytometry and ELISA). Certain B cell culture experiments (Suppl. Fig. 5) were partial replicates of explicitly replicated experiments (Fig. 5) and therefore not repeated further.
Randomization	Randomization was not relevant to this study as covariates (e.g sex, age of animals, cell concentrations in culture) were explicitly matched between experimental and control groups.
Blinding	Blinding was not relevant to this study, as analyses comparing different strains or treatments were done using unilateral cut-offs for each experiment (e.g flow cytometry gates) or had explicit values generated from a machine/computer (cell counts, ELISA ODs).

Reporting for specific materials, systems and methods

We require information from authors about some types of materials, experimental systems and methods used in many studies. Here, indicate whether each material, system or method listed is relevant to your study. If you are not sure if a list item applies to your research, read the appropriate section before selecting a response.

Materials & experimental systems

n/a	Involved in the study	
☐	☒	Antibodies
☒	☐	Eukaryotic cell lines
☒	☐	Palaeontology and archaeology
☐	☒	Animals and other organisms
☒	☐	Clinical data
☒	☐	Dual use research of concern
☒	☐	Plants

Methods

n/a	Involved in the study	
☒	☐	ChIP-seq
☐	☒	Flow cytometry
☒	☐	MRI-based neuroimaging

Antibodies

Antibodies used	The following antibodies or fluorescent dyes/antigen conjugations were used for staining of murine cells ex vivo all following provider's/manufacturer's protocol: Fc receptor block with anti-CD16/32 (5 mg/ml for 20 min on ice) and Live/dead Fixable Aqua (Thermo Fisher, L34957), HA-PE and HA-APC oligomers (kindly provided by Dr. Frances Lund, UAB), BV786 anti-CD19 (1D3) (BD Bioscience, 563333), APC-eFluor780 anti-CD45R (RA3-6B2) (Thermo Fisher, 47-0452-82), PE-Dazzle 594 anti-CD38 (90) (Thermo Fisher, 741748), BV711 anti-CD24 (M1/69) (BD Bioscience, 563450), BV605 anti-CD138 (281-2) (BD Bioscience, 563147), eFluor450 anti-GL-7 (GL7) (Thermo Fisher, 48-5902-82), PE or PE/Cy7 anti-IRF4 (3E4) (Thermo Fisher, 12-9858-82, 25-9858-82), PerCP-eFluor710 anti-IRF8 (V3GYWCH) (Thermo Fisher, 46-9852-82), eFluor450 anti-Ki67 (SolA15) (Thermo Fisher, 48-5698-82), FITC anti-IgM (331) (in-house), and BV650 anti-IgD (11-26c.2a) (Biolegend, 405721). For a non-B cell "dump", the following antibodies on AlexaFluor 700 were used: anti-CD90.2 (Thy1.2) (Biolegend, 105320), anti-CD4 (GK1.5) (Thermo Fisher, 56-0041-82), anti-CD8a (53-6.7) (Thermo Fisher, 56-0081-82), anti-Gr-1 (Thermo Fisher, 56-5931-82), anti-CD11b (M1/70) (Thermo Fisher, 56-0112-82), anti-NK1.1 (Thermo Fisher, 56-5941-82), anti-F4/80 (BM8) (Thermo Fisher, 56-4801-82). The Foxp3 Staining Buffer Set (Thermo Fisher) was used for fixation and permeablization of cells for staining of transcription factors according to manufacturer's protocol. For cytoplasmic only staining, Cytofix/cytoperm buffer set (BD Biosciences) was used according to manufacturer's protocol. For phospho-flow, APC anti-p-Syk (moch1ct) (Thermo Fisher, 17-9014-41), PerCP-eFluor710 anti-p-p38 (4NIT4KK) (Thermo Fisher, 17-9078-42), PE/Cy7 anti-p-mTOR (MRRBY) (Thermo Fisher, 25-9718-41), and PE anti-p-p65 (B33B4WP) (Thermo Fisher, 46-9863-42) were stained according to manufacturer's protocol. All reagents were titrated prior to use to identify dilutions that gave the highest differential fluorescence intensity between the negative and positive cell fraction using mouse spleen, bone marrow or peritoneal cavity wash out cells, as appropriate. Dilutions varied between reagents and reagent lots but typically fell between 1:25 and 1:400. Higher concentrated reagents (mostly those made in-house) were kept prediluted at a concentration that allowed a 1:200 dilution at use.
Validation	All antibodies were purchased from companies well recognized and established for antibody manufacturing and sale. All antibodies were validated by the manufacturer for use in their respective assays and against their listed, cognate antigen. All statements and citations of validation can be found on the product website from each company. Supplemental validation of antibodies for flow cytometry was done using FMO controls, complimentary co-stains with antibodies against a marker found on previously validated cell type, and non-fixed/permeablized cells for antibodies targeting intra-cellular targets. For ELISPOT, anti-Ig antibodies were tested for specificity on purified murine antibodies of different isotypes and non-murine antibodies/serum for cross-adsorption by manufacturer. For c-Rel ELISA/flow, specificity was validated using a c-Rel knockout (Shokhirev et al., 2015, Molecular Systems Biology).

Animals and other research organisms

Policy information about studies involving animals; ARRIVE guidelines recommended for reporting animal research, and Sex and Gender in Research

Laboratory animals	Mice. Male and female 8- to 12-wk-old C57BL/6 (WT; CD45.2 #000664), B6.SJL-Ptprca Pepcb/BoyJ (CD45.1, #002014), B cell–deficient (μMT) mice (#002288), as well as TNFAR1/2 KO (#005540), IFN-gamma KO (#002287), IL-12R KO (#003248), CD19-Cre IFNAR KO, IL-1R KO (#028398), TLR3 KO (#005217), TLR4 KO (#029015), TLR7 KO (#008380) were commercially obtained (The Jackson Laboratories). Breeding pairs of MyD88/TRIF DKO and TLR2/4/unc93b TKO mouse strains were gifts from Dr. Barton (UC Berkeley). Breeding pairs of S100A9 KO mice were a kind gift of Dr. Rafatellu (UC San Diego). Breeding pairs of CD19-Cre IFNAR KO were from Dr. Jason Cyster (UCSF). All mice were housed in SPF housing in ventilated filtertop cages with food and water ad libitum. Euthanasia was done by exposuring mice to C02. All studies involving mice were conducted in strict compliance with and after approval of protocols by the UC Davis Institutional Animal Care and Use Committee.
Wild animals	This study did not involve wild animals
Reporting on sex	Both male and female mice were used for experiments. We did not observe significant differences based on sex and do not report data binned by sex.
Field-collected samples	This study did not involve field-collected samples
Ethics oversight	All studies involving animals were conducted in adherence to protocols approved by the Institutional Animal Care and Use Committee of the University of California Davis.

Note that full information on the approval of the study protocol must also be provided in the manuscript.

Flow Cytometry

Plots

Confirm that:

☒ The axis labels state the marker and fluorochrome used (e.g. CD4-FITC).

☒ The axis scales are clearly visible. Include numbers along axes only for bottom left plot of group (a 'group' is an analysis of identical markers).

☒ All plots are contour plots with outliers or pseudocolor plots.

☒ A numerical value for number of cells or percentage (with statistics) is provided.

Methodology

Sample preparation	Cells were extracted from mice and mechanically sheared mouse tissue and filtered through a 70-micron mesh to generate a single-cell suspension of a buffer of 2% NCS, 1 mM EDTA in KDS-BSS. Tissue collection and processing occurred on ice (4C) at all times. Cells were centrifuged (524 rcf, 4C) and re-suspended to a concentration of 2.5E7 per ml before staining commenced at volumes of 50 or 75 microlitres.
Instrument	BD LSR Fortessa, BD FACSymphony, BD FACS Aria
Software	FACS Diva, Flowjo
Cell population abundance	For negative enrichment of B-2 cells, purity was determined by CD19+ cells in negative fraction, all >98%.<br>For cell sorting of EF PBs and pooled non-EF PBs for influenza-specific ELISPOT, B cells were gated on dump- CD19/CD45R low (EF) vs high (non-EF), then CD38lo/CD24hi. Pooled "non-EF" had 0% gated EF PBs, while "EF PBs" had >75% gated EF PBs.
Gating strategy	All flow cytometry analysis was first serially gated on the following: FSC-A vs SSC-A, FSC-H vs FSC-A, SSC-W vs SSC-A, live cells, dump-negative cells. B cell sub-populations were gated positive or negative based on 1) FMO staining and/or 2) grouping relative to a complimentary marker (e.g CD19 vs CD45R, CD24 vs CD38, IRF8 vs IRF4).

☒ Tick this box to confirm that a figure exemplifying the gating strategy is provided in the Supplementary Information.
